# Dissection of nanoconfinement and proximity effects on the binding events in DNA origami nanocavity

**DOI:** 10.1093/nar/gkab1298

**Published:** 2022-01-17

**Authors:** Sagun Jonchhe, Shankar Pandey, Christian Beneze, Tomoko Emura, Hiroshi Sugiyama, Masayuki Endo, Hanbin Mao

**Affiliations:** Department of Chemistry & Biochemistry, Kent State University, Kent, OH 44242, USA; Department of Chemistry & Biochemistry, Kent State University, Kent, OH 44242, USA; Department of Chemistry & Biochemistry, Kent State University, Kent, OH 44242, USA; Department of Chemistry, Graduate School of Science, Kyoto University, Sakyo, Kyoto 606-8502, Japan; Department of Chemistry, Graduate School of Science, Kyoto University, Sakyo, Kyoto 606-8502, Japan; Institute for Integrated Cell–Material Science (iCeMS), Kyoto University, Sakyo, Kyoto 606-8501, Japan; Department of Chemistry, Graduate School of Science, Kyoto University, Sakyo, Kyoto 606-8502, Japan; Institute for Integrated Cell–Material Science (iCeMS), Kyoto University, Sakyo, Kyoto 606-8501, Japan; Organization for Research and Development of Innovative Science and Technology, Kansai University, Suita, Osaka 564-8680, Japan; Department of Chemistry & Biochemistry, Kent State University, Kent, OH 44242, USA

## Abstract

Both ligand binding and nanocavity can increase the stability of a biomolecular structure. Using mechanical unfolding in optical tweezers, here we found that a DNA origami nanobowl drastically increased the stability of a human telomeric G-quadruplex bound with a pyridostatin (PDS) ligand. Such a stability change is equivalent to >4 orders of magnitude increase (upper limit) in binding affinity (*K*_d_: 490 nM → 10 pM (lower limit)). Since confined space can assist the binding through a proximity effect between the ligand-receptor pair and a nanoconfinement effect that is mediated by water molecules, we named such a binding as mechanochemical binding. After minimizing the proximity effect by using PDS that can enter or leave the DNA nanobowl freely, we attributed the increased affinity to the nanoconfinement effect (22%) and the proximity effect (78%). This represents the first quantification to dissect the effects of proximity and nanoconfinement on binding events in nanocavities. We anticipate these DNA nanoassemblies can deliver both chemical (i.e. ligand) and mechanical (i.e. nanocavity) milieus to facilitate robust mechanochemical binding in various biological systems.

## INTRODUCTION

**Figure 1.**
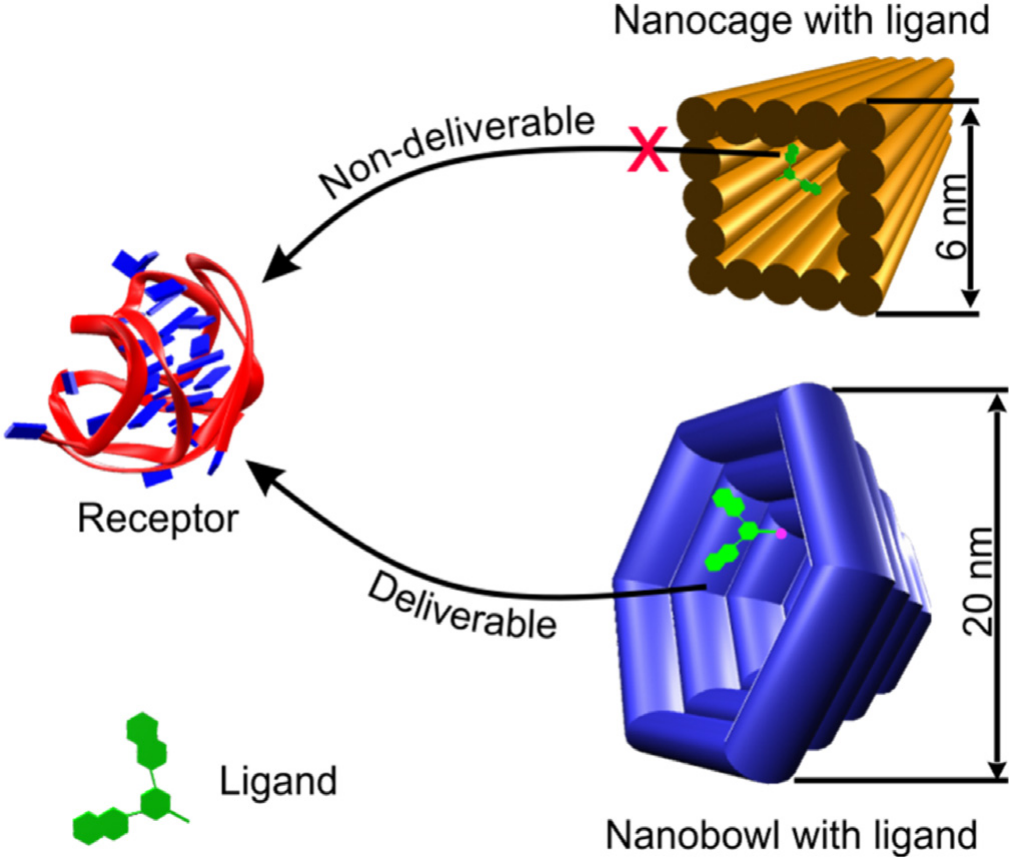
Schematic of deliverable nanoconfinement for enhanced ligand–receptor interactions.

As the first step in many biochemical processes that involve more than one component, binding process has attracted much research attention to modulate subsequent biological processes. Many physiologically relevant chemical and mechanical factors are known to affect binding events. Chemical aspects such as concentrations and properties of buffer components can be varied to shift binding equilibrium from thermodynamic perspective. As a mechanical factor, molecular crowding with a steric effect often increases binding affinities (1). Another mechanical factor to modulate the binding is nanocavity (Figure 1). It has been shown that mixing entropy of unbound components in a nanocavity can be much reduced, which strengthens the binding (2–4). Recently, research has demonstrated that nanocavity can increase the stability of tetraplex DNA structures (5). This effect has been attributed to reduced water activities inside nanocavities. Since water molecules surrounding each binding component are often varied after a binding complex is formed (6,7), it is expected that nanocavity should also exert such a nanoconfinement effect on the binding. Apart from the nanoconfinement effect, nanocavity also provides a proximity effect between binding components, facilitating rebinding of the ligand upon dissociation of the ligand–receptor complex.

Binding can therefore be modulated by chemical or mechanical environments. However, changing the environment brings a global effect to the system in which all processes are affected. To minimize the perturbation, it is desirable to adjust localized environment in immediate proximity of a binding event. Due to its nanometer size, modular nature, and precise modification properties (8), DNA origami structures offer a convenient means to deliver such a localized nanoenvironment to the binding (Figure 1).

In previous investigations, biological molecules such as DNA G-quadruplex have been placed inside a DNA origami nanocage to probe the effect of nanocavity on the folding and unfolding of biomolecules (5). These experiments were performed in optical tweezers to reveal the mechanical information of biomolecular structures. Such an information is physiologically relevant especially in the transcription and replication processes, where motor proteins such as polymerase and helicases can exert load forces onto G-quadruplexes formed along DNA templates (9–11). To investigate the nanocavity effect on the binding from the same mechanical perspective, pyridostatin (PDS) (12) ligands can be added in solution during mechanical unfolding experiments. However, calculation indicated that a ligand concentration of 480 mM is required to populate one molecule inside the nanocage of 5 nm in dimension (Figure 1, top). For many ligands including PDS, such a high concentration is beyond their solubilities.

**Figure 2.**
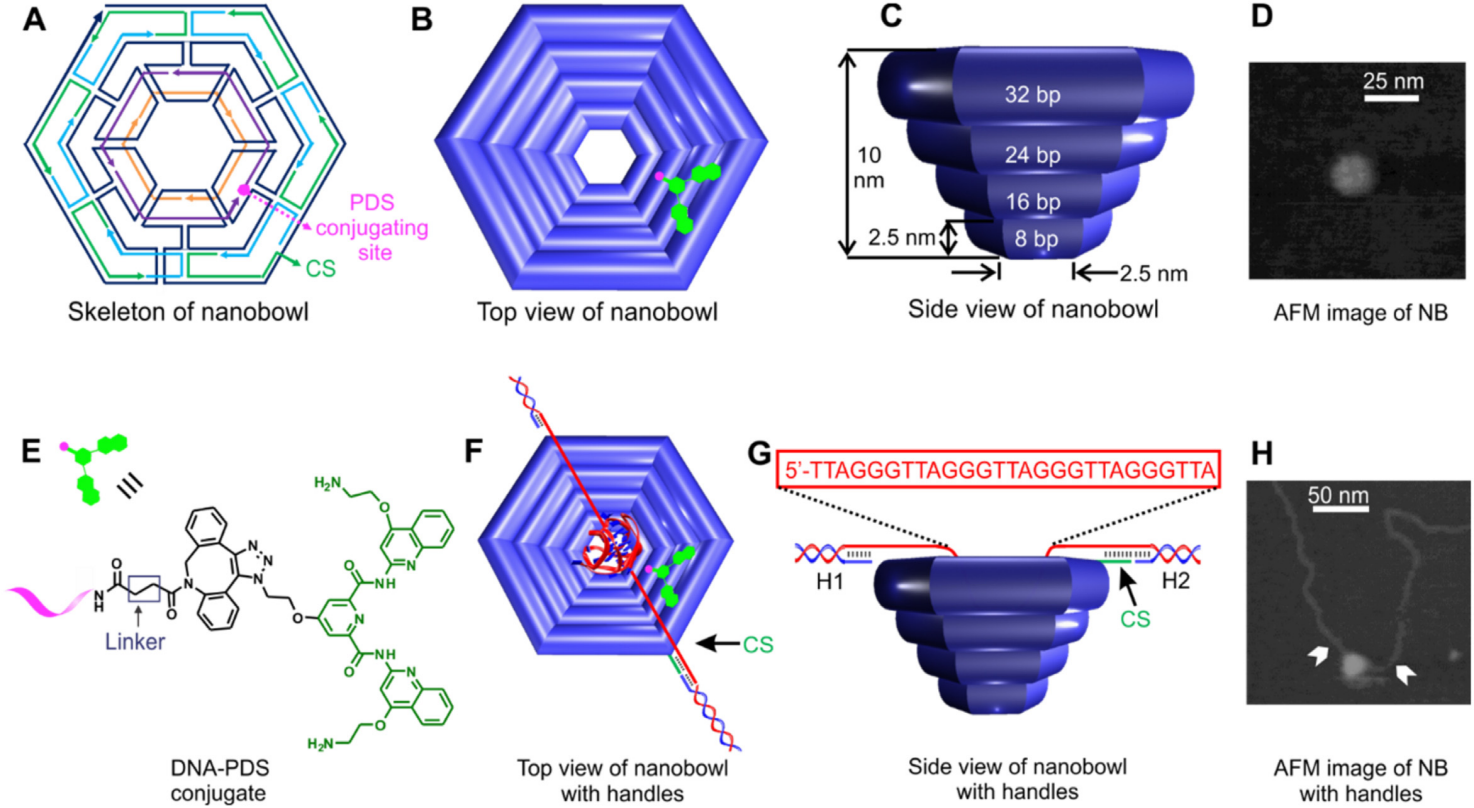
DNA origami nanobowls. DNA strand design (**A**), top view (**B**), side view (**C**) and AFM image (**D**) of nanobowls. Attachment location of the DNA-conjugated pyridostatin (PDS, green) ligand (**E**) is depicted by the pink dot at the inner wall of the nanobowl (B&F). CS designates the connection site for the duplex DNA strand. Top view (**F**), side view (**G**) and AFM image (**H**) of the nanobowls attached to two 2520-bp duplex DNA strands. G-quadruplex sequence is shown in (G).

To tackle this accessibility issue, we designed a bowl-shaped DNA origami in which one portal is open to the solvent while the other tapers to a closure (Figure 2). We placed a telomeric G-quadruplex close to the opening portal of the nanobowl. This allowed the binding of the G-quadruplex to the PDS ligand either tethered inside the nanobowl or freely accessible in solution. Since the binding between the G-quadruplex and the ligand occurs inside the nanobowl, this construct allows us to dissect, for the first time, relative contributions of the proximity effect and the nanoconfinement effect to the binding events in nanocavities.

## MATERIALS AND METHODS

### Materials

All the chemicals, unless specified, were purchased either from VWR (www.vwr.com) or Nacalai Tesque (www.nacalai.com). All the oligos modified with biotin, digoxigenin, amine and PEG linker were obtained from Japan Bio Services. Dibenzocyclooctyne-N-hydroxysuccinimidyl ester was purchased from Sigma Aldrich. Azide modified pyridostatin was prepared as described in literature (13). The streptavidin or anti-digoxigenin coated polystyrene beads were purchased from Spherotech.

### Mechanical unfolding experiments in optical tweezers

**Figure 3.**
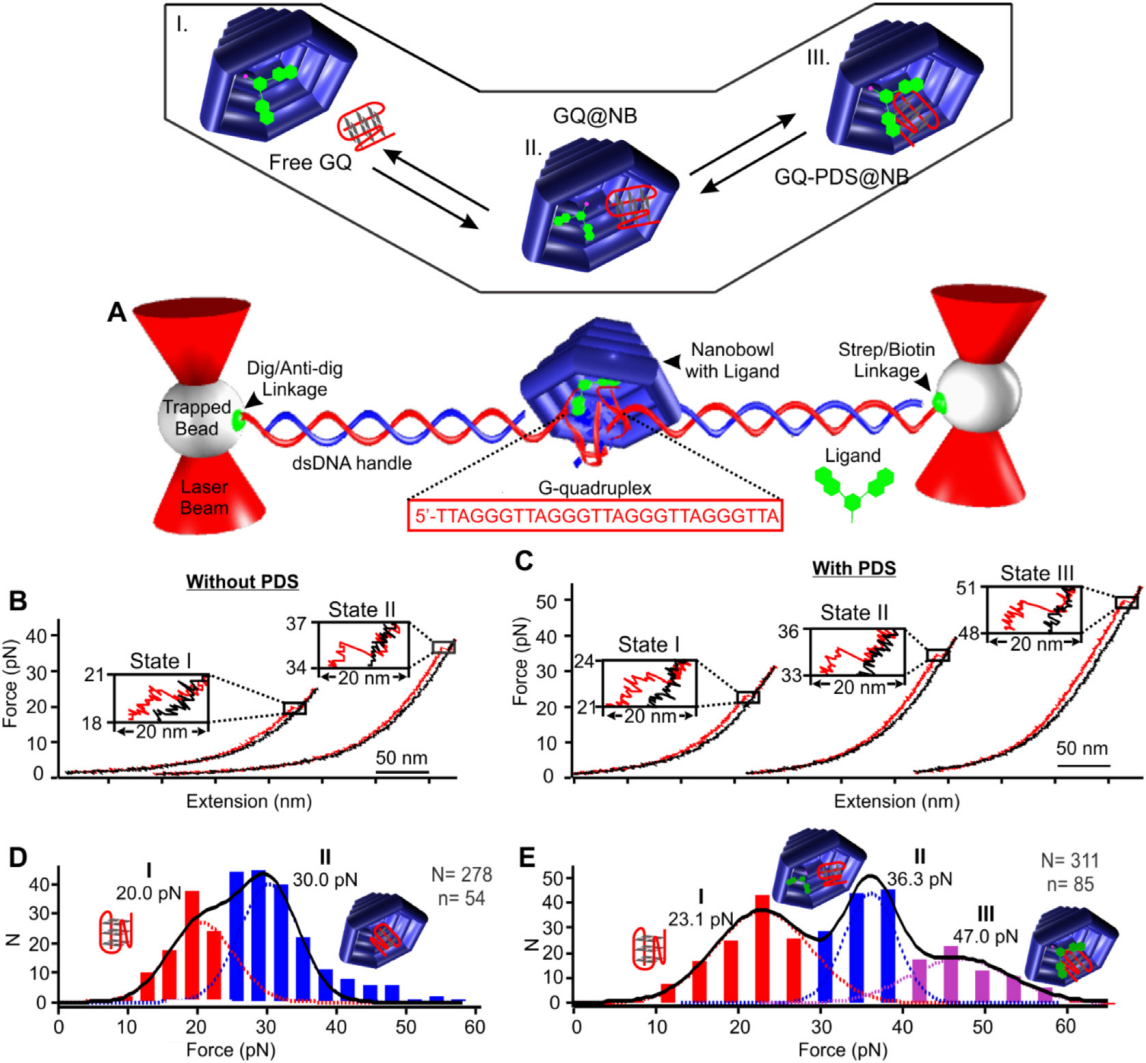
Interaction of the PDS ligand and the telomeric G-quadruplex (GQ) in a nanobowl. (**A**) Mechanical unfolding of the telomeric G-quadruplex inside a nanobowl that contains the PDS ligand. Top inset shows different binding states between the GQ and the PDS. State I, GQ outside the nanobowl (free GQ). State II, GQ inside the nanobowl without binding to the ligand (GQ@NB). State III, GQ bound with PDS inside the nanobowl (GQ-PDS@NB). Stretching (red) and relaxing (black) force–extension (*F*–*X*) curves of the GQ inside the nanobowl without (**B**) and with (**C**) the PDS ligand attached. (**D**) Unfolding force histogram of the GQs in presence of the nanobowl without ligands. (**E**) Unfolding force histogram of the GQ in presence of the PDS ligand attached to the nanobowl. Mechanical experiments were performed in a 20 mM Tris buffer (pH 7.8) supplemented with 10 mM MgCl_2_, 100 mM KCl and 1 mM EDTA at 23°C. *N* and *n* represent the numbers of unfolding events and molecules, respectively.

First, the construct was immobilized on the surface of streptavidin-coated bead by incubating 0.5 μl of the biotin labeled DNA construct with the streptavidin-coated bead to form the streptavidin/biotin linkage (Figure 3). The streptavidin-coated bead with immobilized DNA construct and anti-digoxigenin-coated beads were flowed into top and bottom channels of a three-channel microfluidic chamber, respectively. The beads were flowed into the middle channel of the microfluidic chamber via two micropipettes (i.d.: 25 μm, King Precision Glass, Claremont, CA). Two 1064 nm laser beams in a custom-made dual-trap laser tweezers were used to trap two beads separately (14,15). The DNA tether was formed between two optically trapped beads via digoxigenin/anti-digoxigenin and biotin-streptavidin linkages, which were formed by bringing the two beads closer. This was achieved using a steerable mirror that control the laser beam to trap one of the beads. The force versus extension (*F*–*X*) traces were recorded at 1000 Hz using a Labview program by stretching and relaxing the tether at ∼5.5 pN/s loading rate (in 10–30 pN range) using the same steerable mirror. The experiments were carried out in 20 mM Tris (pH 7.8) buffers supplemented with 10 mM MgCl_2_, 100 mM KCl and 1 mM EDTA at the room temperature.

### Preparation of pyridostatin-attached DNA

The PDS ligand was linked with one of the DNA staples (CBA-3, Supplementary Table S3) using click chemistry (Supplementary Figure S3) (16). First, for the synthesis of DNA-DBCO (Supplementary Figure S3), a 100 μl solution of 40 μl of DNA-NH_2_ (100 μM), 10 μl of DBCO-sulfo-NHS-Ester (5 mM DMF solution) in 0.1 M sodium carbonate buffer (pH 9.0)/30% DMF was incubated at 30°C for 3 h. After the reaction, the mixture was purified by a reversed-phase HPLC using a linear gradient of 2–40% acetonitrile (25 min) with 20 mM ammonium formate. The purified product was lyophilized and dissolved in a 10 mM Tris buffer (pH 7.6). Secondly, for the synthesis of DNA-PDS, a 20 μl solution of 5 μl DNA-DBCO (32 μM), 3 μl azido-PDS (0.1 mM DMF solution) in 0.1 M Tris–HCl (pH 8.0) and 30% DMF was incubated at 30 °C for 3 h. After the reaction, the mixture was purified by a reversed-phase HPLC using a linear gradient of 2–50% acetonitrile (25 min) with 20 mM ammonium formate. The purified product was lyophilized and dissolved in 10 mM Tris buffer (pH 7.6).

### Synthesis of the nanobowl/nanobowl-PDS that contains a G-quadruplex hosting DNA fragment

The DNA nanobowl structures (Figure 2) were designed using the protocol described elsewhere (8). In short, the DNA scaffold was prepared by ligating 5 different strands (Supplementary Table S1) and purified with denaturing PAGE gel, forming the 500-nt scaffold (Supplementary Figure S1). The 25 nM of scaffold DNA was isothermally assembled with DNA staples (1.5 eq), PDS staples (for nanobowl-PDS) (1 eq), and capture strand (see Supplementary Table S3 for DNA sequences) along with the G-quadruplex containing strand (1.5 eq) (Supplementary Figure S2 and Supplementary Supplementary Table S2) from 85°C to 15°C at the rate of −1 °C/min and then 65°C to 15°C at the rate of −0.5 °C/min, resulting in the formation of the nanobowl with G-quadruplex containing strand (Supplementary Figure S4). Subsequently, the construct was annealed with equivalent concentration of two dsDNA handles (Supplementary Figure S5).

### PDS titration experiment

**Figure 4.**
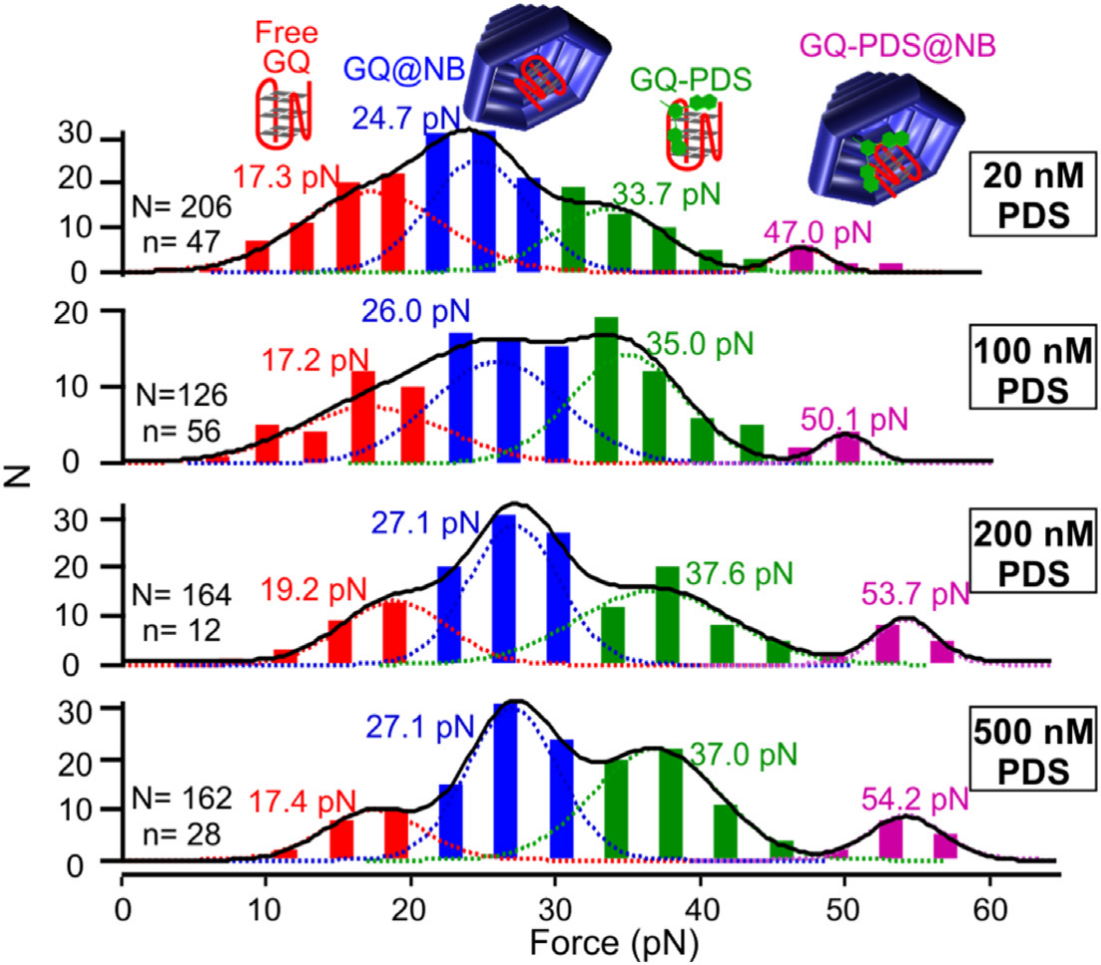
Mechanical stabilities of telomeric G-quadruplexes (GQs) attached to nanobowls (NBs) at different concentrations (20, 100, 200 and 500 nM) of free PDS ligand. Red, blue, green and purple colors indicate free GQ, GQ inside nanobowl (GQ@NB), GQ bound with PDS (GQ-PDS), and GQ bound with PDS inside nanobowl (GQ-PDS@NB), respectively. N and n represent numbers of unfolding events and molecules, respectively.

**Figure 5.**
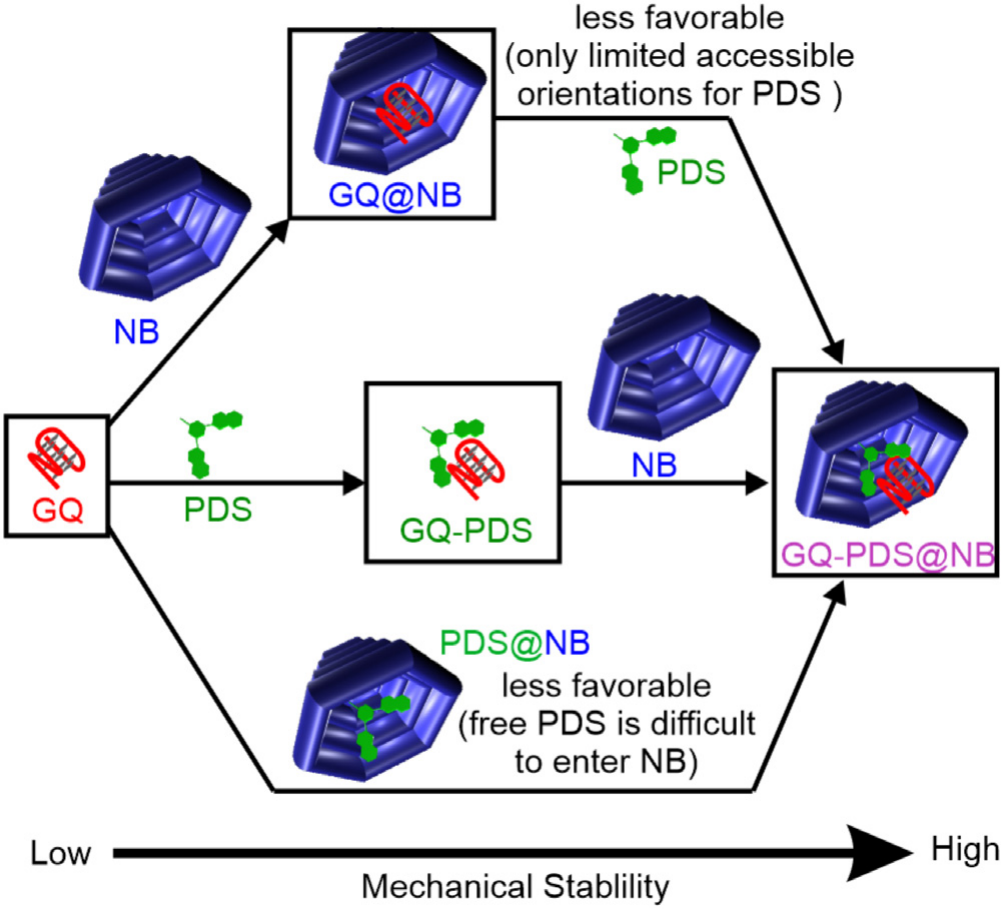
Association pathways of the human telomeric G-quadruplex with DNA nanobowl and free PDS in solution.

G-quadruplex attached to the nanobowl construct was titrated with different concentrations (20, 100, 200 and 500 nM) of free PDS in the pH 7.8 Tris buffer (10 mM MgCl_2_, 100 mM KCl and 1 mM EDTA). The mechanical unfolding of the telomeric G-quadruplex in the nanobowl with different concentrations of PDS was performed according to the procedure described in the section above. The PDS titration resulted in various unfolding force populations (Figures 4 and 5, Table 1).

**Table 1.** Population percentage of unfolding the G-quadruplex (GQ) attached to the nanobowl (NB) in presence of free PDS and nanobowl-immobilized PDS

Sample	Free GQ	GQ@NB	GQ-PDS	GQ-PDS@NB
No ligand	43%	57%	—	—
20 nM PDS*	37%	36%	23%	4%
100 nM PDS*	24%	38%	34%	4%
200 nM PDS*	21%	38%	33%	8%
500 nM PDS*	13%	39%	39%	9%
Immobilized PDS	48%	26%	—	26%

PDS* indicates freely flowing PDS. ‘@’ means ‘inside’.

## RESULTS AND DISCUSSION

### Nanobowl DNA origami attached with a G-quadruplex forming sequence

The DNA nanobowl was designed by using the CadNano program (see Figure 2, Supplementary Figures S1, S4 and S5 in SI for details) (17). AFM images confirmed the formation of nanobowl origami structures (Figure 2D). We used single-molecule force spectroscopy (18–20) to investigate the binding between the G-quadruplex and PDS in the nanobowl. To this end, a human telomeric G-quadruplex forming sequence, 5′-TTA(GGGTTA)_4_TTA, was attached to the two 2520-bp duplex DNA handles (Figure 2 and Supplementary Figure S2), which were separately tethered to two optically trapped polystyrene beads (see Figure 3A and SI for details). A DNA nanobowl was tethered to the dsDNA handle near the G-quadruplex forming sequence, whereas the PDS was immobilized inside the nanobowl close to the opening portal by click chemistry coupling (Figure 2E and Supplementary Figure S3) (16,21). Such a design not only allowed the binding to occur inside nanobowl, it also facilitated the recapture of the bound complex inside nanobowl after disassembly of the G-quadruplex-PDS complex by mechanical force. AFM image demonstrated successful preparation of the construct with long DNA pulling handles (Figure 2H, Supplementary Figures S6 and S7).

Force-ramping experiments were performed to evaluate the binding strength from the perspective of mechanical stabilities in a 20 mM Tris buffer (pH 7.8) supplemented with 10 mM MgCl_2_, 100 mM KCl and 1 mM EDTA at 23 °C (22). By moving one trapped bead away from the other (Figure 3), the tension inside the dsDNA handle increased until the G-quadruplex was mechanically unfolded, which was manifested as a rupture event in Figure 3B. Previously, it was found that the tensile force to unfold free telomeric G-quadruplex was around 20 pN (22), whereas it was 39 pN for the G-quadruplex inside the nanocage (5). Similar rupture force populations were observed (20 and 30 pN) during mechanical unfolding of the G-quadruplex in presence of a nanobowl without PDS ligand (Figure 3D), suggesting that the two force populations correspond to a nanobowl-confined G-quadruplex (GQ@NB, 30 pN) and a free G-quadruplex (Free GQ, 20 pN) in equilibrium. Compared to the 39 pN unfolding force for the same G-quadruplex inside the nanocage (5), the reduced unfolding force (30 pN) observed here likely reflects the fact that the nanobowl does not provide a full enclosure to the G-quadruplex (Figure 2). This geometry also explains the presence of the free G-quadruplex (20 pN) since the structure can readily move outside the nanobowl (Figure 2). Change-in-contour-length (Δ*L* = 8 nm) of each population matched with that expected for telomeric G-quadruplex (see Supplementary Figure S8 and SI for calculation), confirming the assignment of these unfolding features. When we repeated the experiments with a scrambled sequence (5′-GTA GTG TGA TGA GTG TAG TGT GTA GTG) inside nanobowl, we found that unfolding features for folded structures were significantly reduced (Supplementary Figure S9, see Supplementary Figure S7 for an AFM construct image), which corroborated our assignments. It is noteworthy that G-quadruplex may assume diverse mechanical structures when probed by smFRET (23), comparison of change-in-contour-length (Supplementary Figure S8) with those of known G-quadruplex conformations (5) suggests a hybrid-1 topology for the G-quadruplex inside nanobowl.

### Nanocavity drastically increased the binding between an immobilized PDS and G-quadruplex inside the DNA nanobowl

Next, we repeated G-quadruplex unfolding experiments in presence of the nanobowl that contained an immobilized PDS ligand (see Figure 3C for force–extension curves). Notably, we observed three rupture force species located at 23, 36 and 47 pN, respectively. Again, Δ*L* (∼8 nm) measurement for each species matched with that expected for the G-quadruplex (see Supplementary Figure S8 and SI). Comparison of the unfolding forces obtained by above mechanical unfolding experiments without PDS (Figure 3D versus E) suggests that the 23 pN and 36 pN populations are G-quadruplexes outside nanobowl (Free GQ) and inside the nanobowl without binding to the PDS (GQ@NB), respectively. The population with the highest rupture force (47 pN) suggests that the G-quadruplex is bound with the PDS inside nanobowl (GQ-PDS@NB). It has been found previously that PDS bound G-quadruplex has increased mechanical stability (41 pN) (22) with respect to free G-quadruplex. Combined with increased mechanical stability of the G-quadruplex inside nanocage, this indicates that the 47 pN unfolding force population belongs to the G-quadruplex bound with PDS inside nanobowl (GQ-PDS@NB).

After these assignments, using a Hess-like cycle (24) (see Supplementary Figures S10 and S11), Δ*G*_GQ-PDS@NB(dissociation)_ was calculated by the expression, Δ*G*_GQ-PDS@NB(dissociation)_  *= –*Δ*G*_GQ-PDS@NB(binding)_  *=*  *–*(Δ*G*_GQ(unfold)_  *–* Δ*G*_GQ-PDS@NB(unfold)_), where Δ*G*_GQ(unfold)_ (12 (−0.8) kcal/mol, value in parenthesis indicates bias (see Supplementary Figure S10 for detailed deconvolution of different populations and the calculation of the Δ*G* and bias)) and Δ*G*_NB-PDS@NB(unfold)_ (27 (1.3) kcal/mol) are the free energy change of unfolding G-quadruplex without PDS outside nanobowl and that with bound PDS ligand inside nanobowl respectively. The ligand stabilized G-quadruplex inside the nanobowl was found to be more stable with 15 kcal/mol lower in free energy compared to the G-quadruplex in dilute buffer condition. Next, we estimated the dissociation constant (*K*_d_) as 10 pM by the expression, Δ*G*_GQ-PDS@NB(dissociation)_  *= –RT* ln(*K*_d_), where *R* is the gas constant, *T* is absolute temperature, and Δ*G*_GQ-PDS@NB(dissociation)_ is the change in the free energy of dissociating PDS-bound G-quadruplex inside nanobowl. Although our change in free energy of G-quadruplex unfolding calculated from non-equilibrium Jarzynski equation (25) is consistent with experimental measurements of 4.4–14.8 kcal/mol (26–28), it is possible that overestimation exists for the change in free energy calculation in the nanobowl. Therefore, the *K*_d_ represents a lower limit estimation for the binding constant. Nevertheless, from the mechanical force measurement (*F*_unfold(GQ-PDS@nanobowl)_, 47.0 pN > *F*_unfold(GQ-PDS)_, 36.3 pN), the trend of increased binding is solid inside nanobowl.

It is significant that *K*_d_ of the G-quadruplex and PDS complex inside nanobowl is ∼50 000 times tighter than free solutions (490 nM) (22). The strikingly large increase in the binding affinity indicates that nanocavity provides an effective means to strengthen the ligand binding to the G-quadruplex. Unlike the ligand binding that involves chemical interactions such as intermolecular forces, the nanocavity is largely mechanical in nature. The nanospace surrounding the bound complex restricts the possibility of different conformations that can be assumed by the complex compared to corresponding free components, which facilitates the binding by reducing the entropic penalty. Such a nanoconfinement effect can also be mediated by the chemical activity of water molecules inside the nanocavity (29). Apart from the nanoconfinement effect, the nanocavity also introduced a constrict space in which ligand and receptor stay in proximity. Such a proximity effect allows rapid reassociation of the binding components upon their disassembly, effectively increasing the binding affinity. Given the largely mechanical nature of the nanocavity and the chemical nature of the ligand binding, we refer the binding process in nanocavity as mechanochemical binding.

### Quantification of proximity and nanoconfinement effects on the binding of GQ and PDS inside nanoconfinement

To quantify the relative contributions of the proximity effect and the nanoconfinement effect to the much-stabilized G-quadruplex (GQ)–PDS binding complex in the nanobowl, we flowed a series of PDS containing solutions to a nanobowl anchored telomeric GQ (no PDS was immobilized in the nanobowl). We anticipated the large opening in the nanobowl facilitated the entering as well as leaving of free PDS, which eliminated the proximity effect leading to the rapid reassociation of the GQ-PDS binding complex. As shown in Figure 4, addition of the PDS introduced a total of four species in the unfolding force histograms. Based on the values of the unfolding force as discussed above, the population close to 20 pN was assigned as the free G-quadruplex outside the nanobowl (free GQ) (22). The 24–27 pN population was likely the ligand-free GQ in the nanobowl (GQ@NB) whereas the 33–37 pN structure was ascribed to the PDS-bound GQ outside nanobowl (GQ-PDS). These two assignments were based on the observations that the same telomeric GQ bound with PDS had a higher mechanical force (∼41 pN) (22) than that of the telomeric GQ inside a DNA origami nanocage (∼39 pN) (5) or that of the GQ in the nanobowl discussed above (30 pN, Supplementary Figure 3B&D). Student's t test revealed that GQ@NB has the unfolding force (24–27 pN) indistinguishable with that (30 pN) of the same species in Figure 3D at 99.99% confidence level. It is interesting that the unfolding force of this species is significantly lower than that (36.3 pN) of the state II in Figure 3E. This increased force (36.3 pN) can be explained by more constricted space and therefore, greater nanoconfinement effect on the free G-quadruplex in the state II (Figure 3E) in which the immobilized PDS does not bind to the GQ inside the nanobowl. Finally, the >47 pN population was attributed to the PDS-bound GQ inside the nanobowl (GQ-PDS@NB) since it was conceivable that nanocavity effect and the ligand binding effect had an additive effect on the GQ stability. Given that the proximity effect was not present in these PDS titration experiments, the >47 pN population (GQ-PDS@NB) was likely due to the nanoconfinement effect as a result of the small size of the nanocavity and more ordered water molecules inside the nanocavity (29).

These species allowed a total of three ligand binding and nanobowl encapsulating pathways (Figure 5). The bottom pathway (Free GQ → GQ-PDS@NB) was less likely to occur with respect to the other two pathways (top pathway: free GQ → GQ@NB → GQ-PDS@NB; middle pathway: free GQ → GQ-PDS → GQ-PDS@NB) since the driving force for the PDS@NB formation in the free PDS solution was not as high as the thermodynamically more stable complexes of the GQ@NB or the GQ-PDS. As expected, when concentrations of PDS increased from 0 to 500 nM, a gradual shift of populations to higher unfolding force species was observed. While free GQ and GQ@NB decreased their populations, GQ-PDS and GQ-PDS@NB increased their percentages (Table 1). Such a trend suggested that the middle pathway, Free GQ → GQ-PDS → GQ-PDS@NB, was predominant among three pathways since GQ-PDS was not expected to exist in the top pathway (free GQ → GQ@NB → GQ-PDS@NB).

It is significant that GQ-PDS@NB has a mechanical stability (Gaussian centers > 47 pN, average 51 pN, Figure 4) higher than the corresponding species in Figure 3 (State III, 47 pN). Due to the fact that both PDS and G-quadruplex were anchored to the nanobowl (Figure 3), more energy was required to align the binding complex (State III) along an optimized interacting orientation, which decreased the mechanical stability of the binding complex. The decreased average mechanical stability in the State III of Figure 3 may be also caused by the proximity effect: the rapid reassociation of a priorly dissociated PDS-GQ pair implied that not enough time was available to adopt the most stable binding conformation. Taken together, the GQ-PDS@NB species (47 pN, State III) in Figure 3 likely reflected the combined proximity effect and nanoconfinement effect, whereas the >50 pN GQ-PDS@NB population in Figure 4 can be attributed to the nanoconfinement effect since PDS can freely enter and leave the nanobowl, which reduces the proximity effect. Fitting the randomly deconvoluted (30) unfolding force histogram in the range of 32–63 pN in Figure 3E therefore allowed to estimate that the proximity effect (∼47 pN) and the nanoconfinement effect (∼54 pN) contribute 78% and 22%, respectively, to the binding between the immobilized PDS and G-quadruplex in the DNA origami nanocavities (Supplementary Figure S12).

When the PDS was not present in the nanobowl, the pure nanoconfinement effect increased the stability of the G-quadruplex as expected (5) (Supplementary Figure 3B and D). To compare the effects of the nanoconfinement and ligand-binding on the mechanical stability of the G-quadruplex, we calculated an apparent dissociation constant of the nanobowl to the G-quadruplex, *K*_d(apparent)_, by the following expression, (Δ*G*_GQ@NB(unfold)_  *–* Δ*G*_GQ(unfold)_) = –*RT*ln(*K*_d(apparent)_), where Δ*G*_GQ@NB(unfold)_ and Δ*G*_GQ(unfold)_ depict the changes in the unfolding free energy of G-quadruplexes within and without nanobowls (see Supplementary Figure S10 for Δ*G* values). The calculation yielded *K*_d(apparent)_ of 561 nM equivalent of the PDS binding to the G-quadruplex, which is similar to the *K*_d_ of the PDS and G-quadruplex complex in free solution (*K*_d_ = 490 nM) (22). This suggests that nanobowl has a comparable effect to stabilize G-quadruplex with respect to the chemical (PDS) binding. As the G-quadruplex can freely diffuse in and out of the nanobowl, such a mechanochemical binding in the nanocavity is expected to be transient. For a sustainable mechanochemical binding effect, therefore, it is desirable to covalently attach a ligand inside the nanocavity to retain the G-quadruplex. The entire construct (the nanobowl with an immobilized ligand, Figure 1) can then be delivered as a mechanochemical binding module to biological targets.

## CONCLUSIONS

In summary, we have demonstrated that nanocavity inside a DNA origami nanobowl assembly increases the binding affinity between the telomeric G-quadruplex and the PDS ligand by >4 orders of magnitude. Such dramatic increase is contributed by the nanoconfinement effect (22%) and the proximity effect (78%). We called such a nanocavity modulated binding as mechanochemical binding to reflect the mechanochemical nature of the binding interaction. Since different functional groups can be incorporated inside this mechanochemical binding module, desired chemical or mechanical nanoenvironments can be delivered to biomolecular targets. These nanoenvironments are expected to exert only localized effect within proximity of a binding event, which avoids unwanted global effects, such as changes in temperature or ionic conditions, on the entire biological system.

## Supplementary Material

gkab1298_Supplemental_File
