# Supplementary material for: Dissection of nanoconfinement and proximity effects on the binding events in DNA origami nanocavity
Source: Nucleic Acids Res. 2022 Jan 17;50(2):697–703. doi: 10.1093/nar/gkab1298 (PMC8789071; doi:10.1093/nar/gkab1298)
Supplement: gkab1298_Supplemental_File [file gkab1298_supplemental_file.docx]

**Supporting Information**

**Dissection of nanoconfinement and proximity effects on the binding events in DNA origami nanocavity**

Sagun Jonchhe^1^, Shankar Pandey^1^, Christian Beneze^1^, Tomoko Emura^2^, Hiroshi Sugiyama^2,3,^*, Masayuki Endo^2,3,4,^*, Hanbin Mao^1,^*

^1^Department of Chemistry & Biochemistry, Kent State University, Kent, Ohio, 44242, USA

^2^Department of Chemistry, Graduate School of Science, Kyoto University, Sakyo, Kyoto 606–8502, Japan

^3^Institute for Integrated Cell–Material Science (iCeMS), Kyoto University, Sakyo, Kyoto 606–8501, Japan

^4^Organization for Research and Development of Innovative Science and Technology, Kansai University, Suita, Osaka 564-8680, Japan

**Table of Contents**

[**1.** **Preparation of scaffold template strand** S3](#_Toc80049345)

[**2.** **Synthesis strategy for the 139-nt single-stranded DNA containing G-quadruplex forming sequence** S5](#_Toc80049346)

[**3.** **Preparation of pyridostatin-attached DNA** S7](#_Toc80049347)

[**4.** **Synthesis of the nanobowl/nanobowl-PDS** S8](#_Toc80049348)

[**5.** **Synthesis of the nanobowl/nanobowl-PDS that contains G-quadruplex hosting DNA fragment** S9](#_Toc80049349)

[**6.** **Synthesis of double-stranded DNA handles** S10](#_Toc80049350)

[**7.** **Characterization of the single molecular DNA nanobowl constructs by AFM** S12](#_Toc80049351)

[**8.** **Change-in-contour-length measurement** S13](#_Toc80049352)

[**9.** **Percentage feature formation** S14](#_Toc80049353)

[**10.** **Expected change-in-contour-length (Δ*L*)** S14](#_Toc80049354)

[**11.** **Calculation of the change in free energy of unfolding (Δ*G*_unfold_)** S15](#_Toc80049355)

[**12.** **Hess-like cycle** S16](#_Toc80049356)

[**13.** **Proximity effect versus nanoconfinement effect** S17](#_Toc80049357)

# **Preparation of scaffold template strand**

The 500-nt single-stranded DNA scaffold was prepared by splint ligation of the five pieces of DNA oligonucleotides (Figure S1). T1, T2, T3, T4 and T5 oligos were first phosphorylated by polynucleotide kinase, followed by ligation using T4 DNA ligase assisted by CT-12, CT-23, CT-34 and CT-45 splints (Table S1). Finally, the splint DNA oligos were removed by denatured PAGE to obtain pure 500-nt single-stranded DNA scaffold (Figure S1).


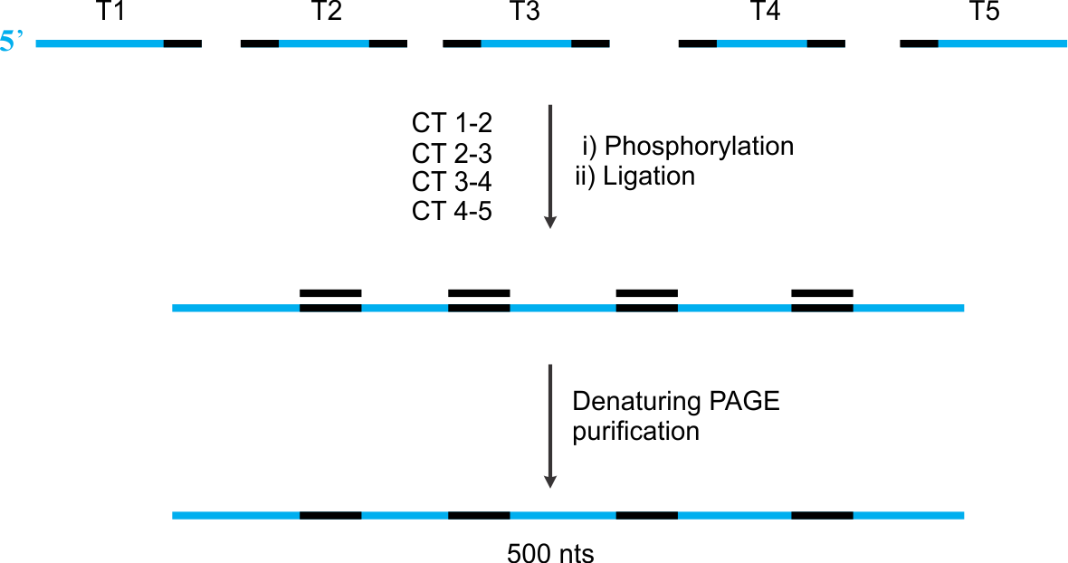


***Figure S1.*** *Flowchart for the preparation of 500-nt DNA scaffold template used for the synthesis of nanobowl (NB).*

***Table S1.*** *DNA staple sequences used for the synthesis of the DNA scaffold template strand of the nanobowl nanoassembly. The black regions in T1, T2, T3, T4, and T5 oligos represent splint recognizing regions. CT-12, CT-23, CT-34 and CT-45 are the splints.*

| **Staple Name** | **Sequence** | **Length** |
| --- | --- | --- |
| T1 | TGATAGACGGTTTTTCGCCCTTTGACGTTGGAGTCCACGTTCTTTAATAGT  GGACTCTTGTTCCAAACTGGAACAACACTCAACCCTATCTCGGGCTATT | 100 |
| T2 | CTTTTGATTTATAAGGGATTTTGCCGATTTCGGAACCACCATCAAACAGGA  TTTTCGCCTGCTGGGGCAAACCAGCGTGGACCGCTTGCTGCAACTCTCT | 100 |
| T3 | CAGGGCCAGGCGGTGAAGGGCAATCAGCTGTTGCCCGTCTCACTGGTGAAA  AGAAAAACCACCCTGGCGCCCAATACGCAAACCGCCTCTCCCCGCGCGT | 100 |
| T4 | TGGCCGATTCATTAATGCAGCTGGCACGACAGGTTTCCCGACTGGAAAGCGG  GCAGTGAGCGCAACGCAATTAATGTGAGTTAGCTCACTCATTAGGCAC | 100 |
| T5 | CCCAGGCTTTACACTTTATGCTTCCGGCTCGTATGTTGTGTGGAATTGTGAGC  GGATAACAATTTCACACAGGAAACAGCTATGACCATGATTACGAATT | 100 |
| CT- 12 | CCT TAT AAA TCA AAA GAA TAG CCC GAG ATA GG | 32 |
| CT- 23 | TTC ACC GCC TGG CCC TGA GAG AGT TGC AGC AAG | 32 |
| CT- 34 | ATT AAT GAA TCG GCC AAC GCG CGG GGA GAG GC | 32 |
| CT- 45 | AAG TGT AAA GCC TGG GGT GCC TAA TGA GTG AG | 32 |

# **Synthesis strategy for the 139-nt single-stranded DNA containing G-quadruplex forming sequence**

The 139-nt single-stranded DNA, which contains a telomeric G-quadruplex motif was synthesized by using splint ligation of two short DNA oligonucleotides (Figure S2 and Table S2). All the DNA oligonucleotides (GQ-1 new and GQ 3* in Figure S2) were first phosphorylated by polynucleotide kinase, followed by ligation using T4 DNA ligase assisted by the splint DNA oligos (complementary 1-3, see Table S2 for sequences). The splint DNA oligos were removed by denaturing PAGE to obtain pure 139-nt single-stranded DNA. The purified 139-nt oligo was finally annealed with the connector strand.


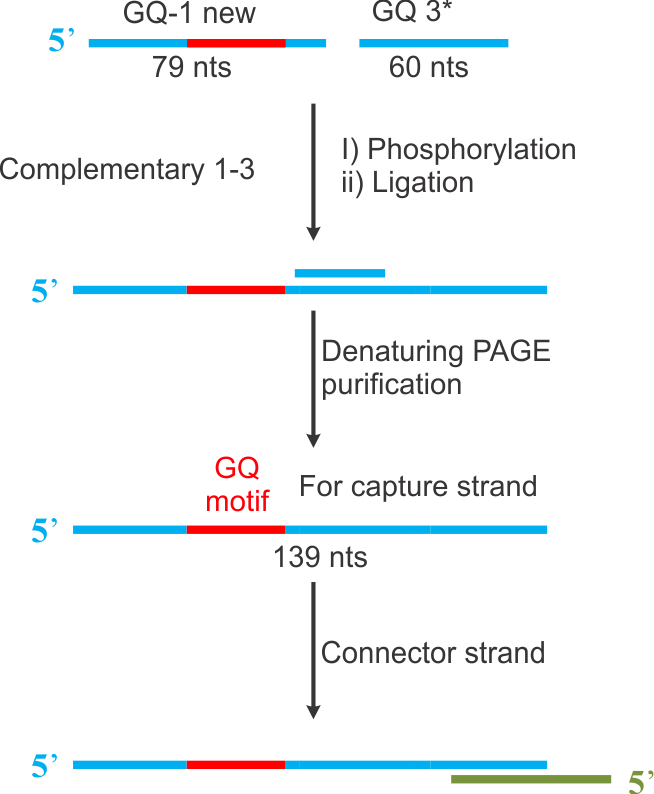


***Figure S2.*** *Flowchart for the synthesis of the 139-nt DNA fragment containing G-quadruplex forming sequence. This fragment passes on top of the portal of the DNA nanobowl.*

***Table S2.*** *DNA sequences for the synthesis of G-quadruplex containing fragment.*

| Oligo name | Sequence | Length |
| --- | --- | --- |
| GQ strand-1 new | TTACCTTTCCCCTCTG-GCAAAACTATTCCGGT–AAAC-TTAGGGTTAGGGTTAGGGTTAGGG TTA -TTTTTTTT-ATTTCTTC | 79 |
| GQ strand 3* | AGGCTTAA-CCATGCATTCCGATTG-CAGC-TTGCATCCATTGCATC-GCTTGAATTGTCCAGC | 60 |
| Complementary 1-3 new | ATGG-TTAAGCCT-GAAGAAAT-AAAA | 24 |
| Connector strand | GCCCTTTGTGGTTCAC-GTAGTGGGTGGCGCCC-GCTGGACAATTCAAGC-GATGCAATGGATGCAA | 64 |

# **Preparation of pyridostatin-attached DNA**


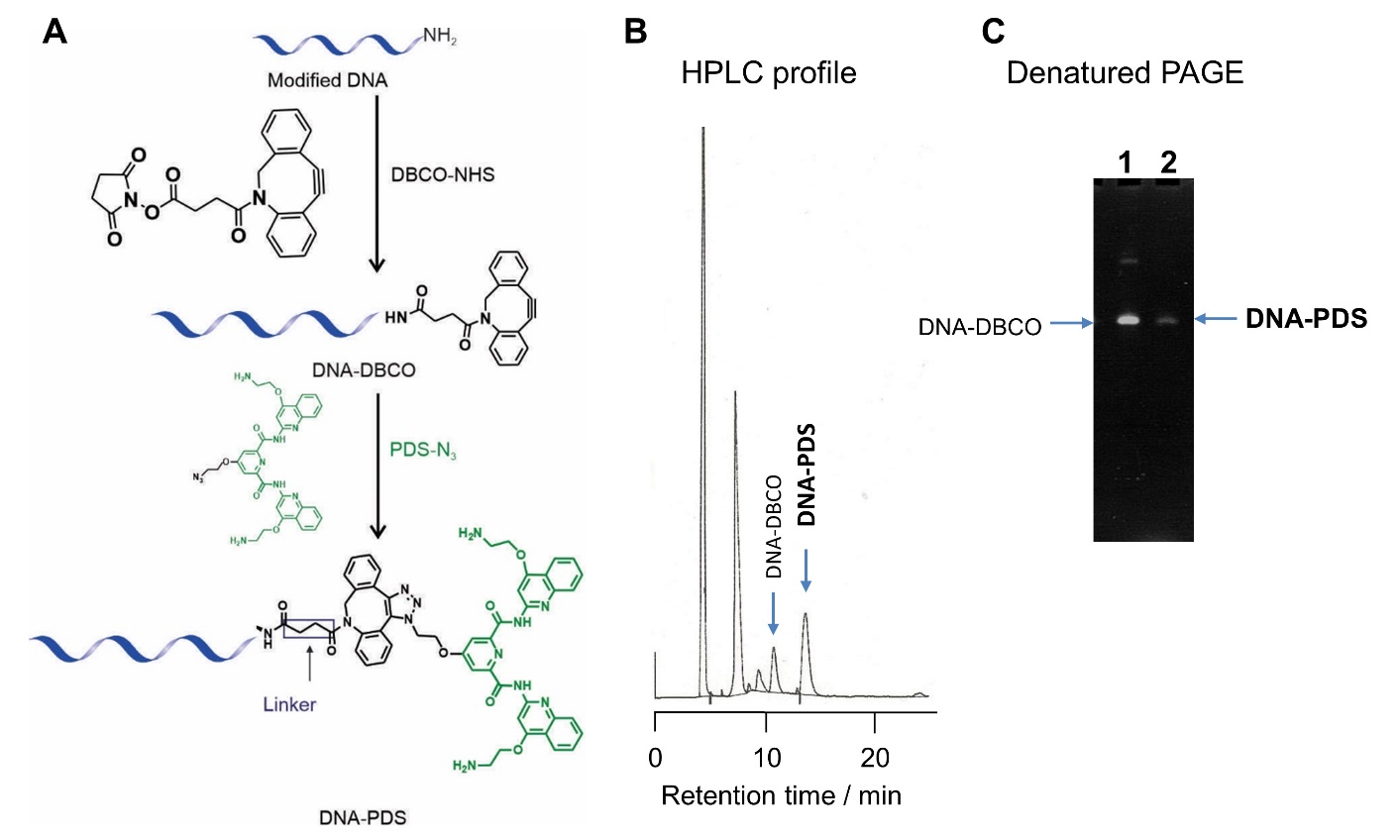


***Figure S3.*** *Flowchart for attachment of the pyridostatin (PDS) with the DNA using click chemistry. (A) Reaction scheme for the synthesis of the DNA-PDS. (B) HPLC profile of the mixture of the DNA-DBCO and PDS-N3 after the reaction. (C) Denatured PAGE (16%, 1×TBE, 8M urea) of DNA-DBCO (lane 1) and DNA-PDS (lane 2) after the HPLC purification.*

# **Synthesis of the nanobowl/nanobowl-PDS**


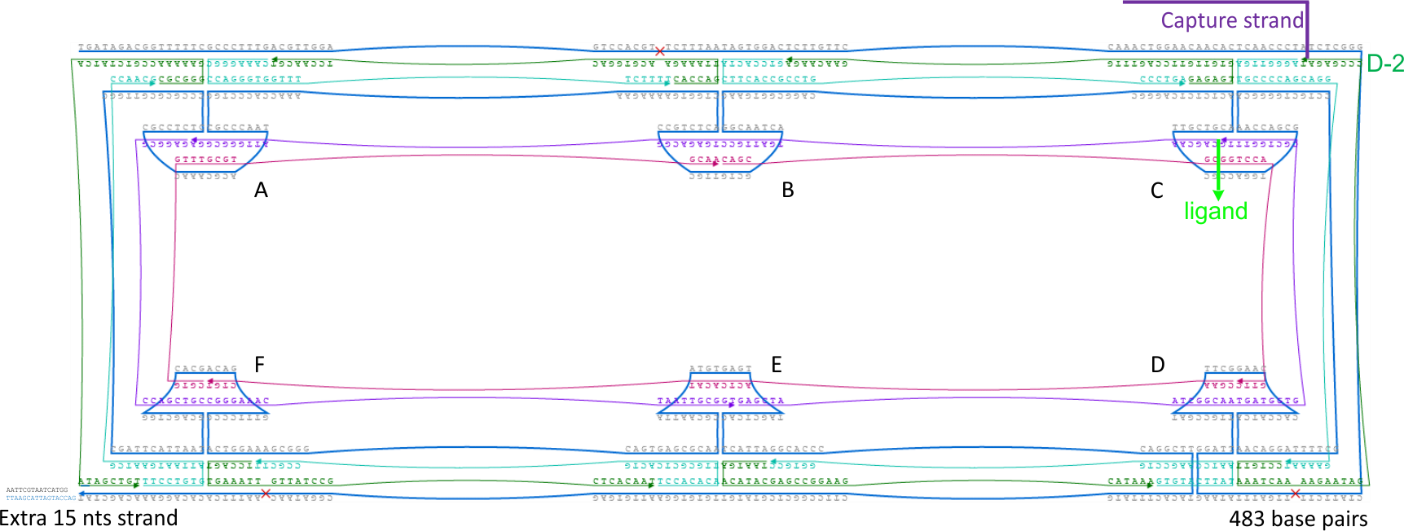
The DNA nanobowl structures were designed using the protocol described elsewhere.(S[1](#_ENREF_1)) In short, the DNA scaffold was prepared by ligating 5 different strands and purified with denaturing PAGE gel, forming 500-nt scaffold (Figure S1). The 25 nM scaffold DNA was isothermally assembled with 20 mM staples (1.5 eq), and PDS staples (for nanobowl-PDS) (1 eq), from 85 ⁰ C to 65 ⁰ C at the rate of -1 ⁰ C and then 65 ⁰ C to 15 ⁰ C at the rate of -0.5 ⁰ C, resulting in formation of the nanobowl or nanobowl with PDS.

***Figure S4.*** *Design of the nanobowl/nanobowl-PDS.* *The caDNAno design of the DNA origami nanobowl and nanobowl with PDS.* ***Ligand*** *and* ***Capture strand*** *represent the connection sites of the PDS ligand (strand CBA-3 in Table S3) and connection with the G-quadruplex containing strand, respectively. The 500-nt strand made by ligation and purification strategy (Figure S1) was used as a template strand.*

# **Synthesis of the nanobowl/nanobowl-PDS that contains G-quadruplex hosting DNA fragment**


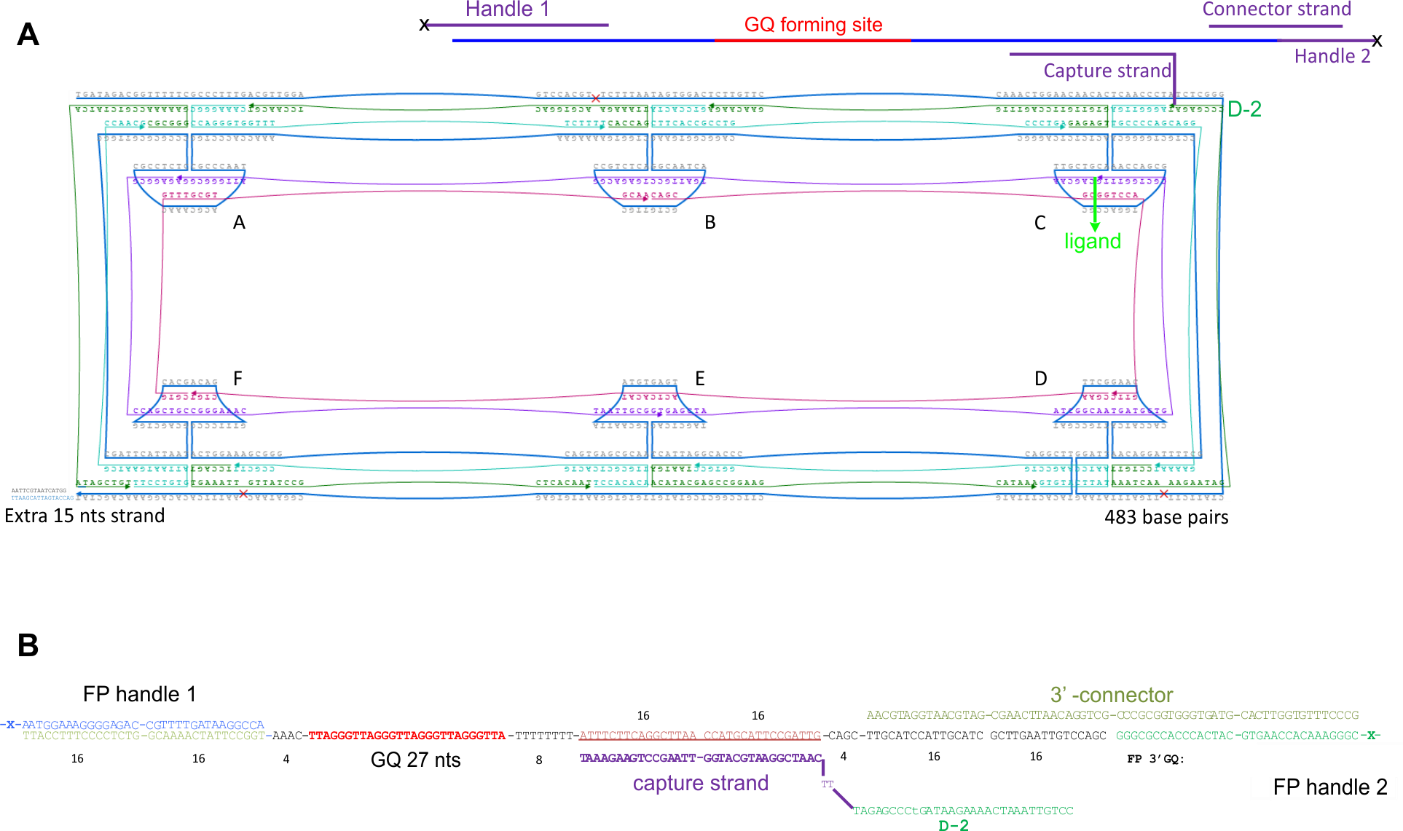


***Figure S5.*** *Design of the nanobowl/nanobowl-PDS with G-quadruplex hosting DNA fragment. (****A****)* *The caDNAno design along with the attachment of G-quadruplex containing strand.* ***Ligand*** *and* ***Capture strand*** *represent the connection sites of the PDS ligand (strand CBA-3 in Table S3) and connection with the G-quadruplex containing strand, respectively. The 500-nt strand made by ligation and purification strategy was used as a template strand. (****B****) Detailed sequence of the 139nt G-quadruplex hosting strand. Tethering site to the nanobowl with capture strand and attachment site to the dsDNA handles are represented. Each number in (B) indicates the length of the nucleotides of a specific fragment.*

***Table S3.*** *DNA staple sequences used for the synthesis of nanobowl/nanobowl-PDS. The small font “t” indicates non-hybridizing nucleotides.*

| **Staple Name** | **Sequence** | **Length** |
| --- | --- | --- |
| A-1 | CAAAGGGCCCAGGGTGGTTTtTCTTTT | 27 |
| B-1 | GTCCACTACTTCACCGCCTGtCCCTGA | 27 |
| C-1 | AGGGTTGATGCCCCAGCAGGtGAAAAT | 27 |
| D-1 | GTGTACTTATAATCCAAGCCTGtGGTGCC | 29 |
| E-1 | TCCACACATTGCGCTCACTGtCCGCTT | 27 |
| F-1 | TTCCTGTGATTAATGAATCGtCCAACG | 27 |
| A-2 | CGCGGGGAAAAACCGTCTATCAtATAGCTGT | 31 |
| B-2 | CACCAGTTAAAGAACGTGGACtTCCAACGT | 30 |
| C-2 | GAGAGTGTGTTGTTCCAGTTTGtGAACAAGA | 31 |
| D-2 | CCTGTTAAATCAAAAGAATAGtCCCGAGAT | 30 |
| E-2 | TAATGAACATACGAGCCGGAAGtCATAAA | 29 |
| F-2 | TCCAGTTGAAATTGTTATCCGtCTCACAAT | 30 |
| AFE-3 | GAGGCGtCCAGCTGCCGGGAAACtTAATTGCGGT | 34 |
| EDC-3 | GAGCTAtATCGGCAATGATGGTGtCGCTGGTTTG | 34 |
| CBA-3 | CAGCAAtTGATTGCCTGAGACGGtATTGGGCGGA | 34 |
| BCD-4 | CAGCtGCGGTCCAtGTTC | 18 |
| DEF-4 | CGAAtACTCACATtCTGT | 18 |
| FAB-4 | CGTGtGTTTGCGTtGCAA | 18 |
| capture strand D-2 | CCTGTTAAATCAAAAGAATAGtCCCGAGATttCAATCGGAATGCA  TGGTTAAGCCTGAAGAAAT | 64 |

# **Synthesis of double-stranded DNA handles**

Two double-stranded 2520 bp DNA handles were prepared by PCR amplification of the pET-26b (+) plasmid. The forward primer of each handle consists of “5'-Connecting (linker) Sequence-O-(CH_2_)_2_-O-CH_2_)_2_-O-(CH_2_)_2_-O-Primer Sequence” (see below). This design introduced a single-stranded 5'-overhang in one end of each handle, which serves as a staple to hybridize with the nanocage. The other end of the biotin handle is labeled with biotin using 5'-biotin modified reverse primers. For the digoxigenin handle, poly-digoxigenin labelling is introduced at the 3′ end after purifying the SacI digested PCR product.

**Primers for biotin-labeled handle**

Forward Primer: 5'-ACC GGA ATA GTT TTG C-CA GAG GGG AAA GGT AA-**X**-CGC CGA TCA ACT GGG TGC CAG CGT

Reverse Primer: 5'-Biotin-GGG TTC GTG CAC ACA GCC CAG CTT

**X** = O-(CH_2_)_2_-O-(CH_2_)_2_-O-(CH_2_)_2_-O

**Primers for digoxigenin-labeled handle**

Forward Primer: 5'- GGG CGC CAC CCA CTA C-GT GAA CCA CAA AGG GC**-X-** CGC CGA TCA ACT GGG TGC CAG CGT

Reverse Primer: 5’- AAA AAA AAG AGC TCG GGT TCG TGC ACA CAG CCC AGC TT

**X** = O-(CH_2_)_2_-O-(CH_2_)_2_-O-(CH_2_)_2_-O

# **Characterization of the single molecular DNA nanobowl constructs by AFM**


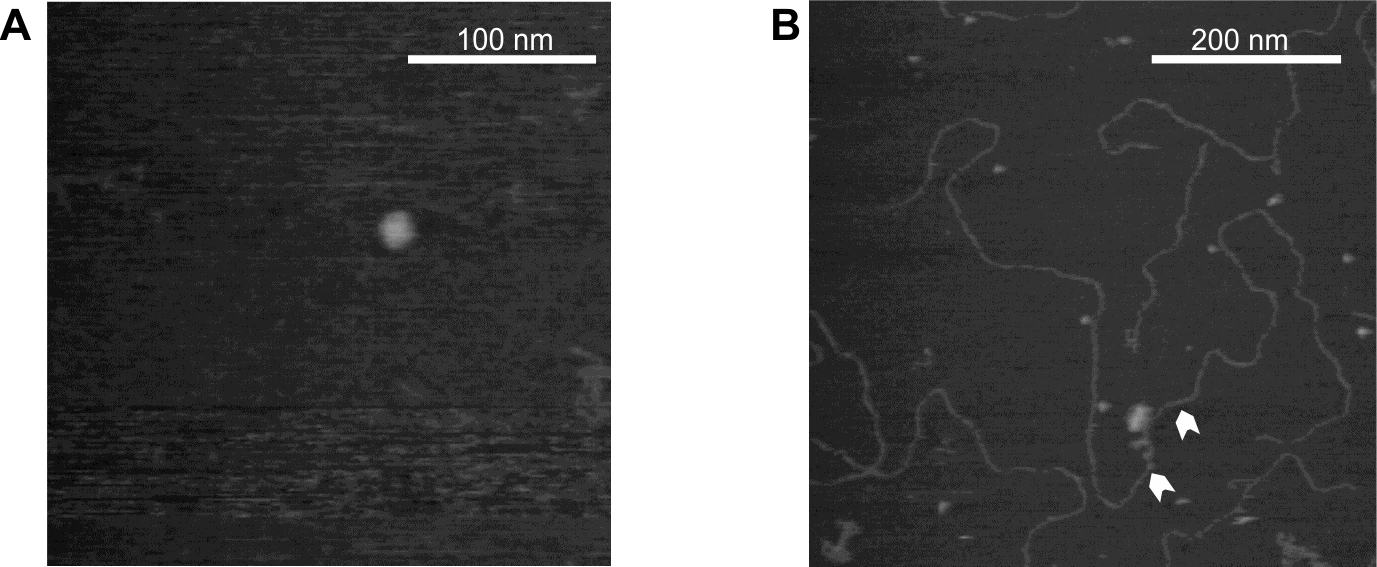
AFM imaging was carried out at the scan rate of 0.2 frames per seconds (fps) from AFM system (Nano Live Vision, RIBM, Tsukuba, Japan) with a silicon nitride cantilever (resonant frequency = 1.0 - 2.0 MHz, spring constant = 0.1 - 0.3 N/m, EBD Tip radius <15 nm, Olympus BLAC10EGS-A2). 2 µL sample was adsorbed on freshly cleaved mica plate [Φ 1.5 mm, pretreated with 0.1% 3-aminopropyl trimethoxysilane (APTES)] for 5 min at room temperature. The adsorbed sample was further washed several times with 20 mM Tris buffer (pH 7.6) containing, 10 mM MgCl_2_ and 1 mM EDTA.

***Figure S6.*** *AFM images of (A) nanobowl and (B) nanobowl with G-quadruplex connected to the DNA handles. The arrowheads indicate the dsDNA handles connected to the nanobowl.*


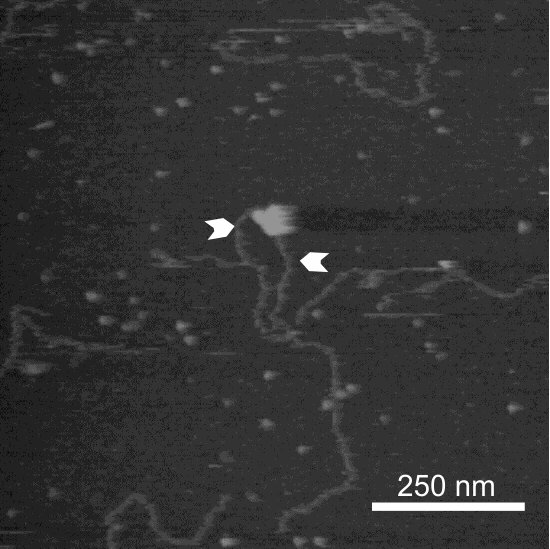


***Figure S7.*** *AFM image of the nanobowl with a scrambled sequence (5′-GTA GTG TGA TGA GTG TAG TGT GTA GTG) connected with the dsDNA handles. The arrowheads indicate the dsDNA handles connected to the nanobowl.*

# **Change-in-contour-length measurement**

Change in extension (Δ*x*) was obtained by measuring the length between two data points flanking the unfolding event at the same force (*F*). Change-in-contour-length (Δ*L*) was obtained from Δ*x* using a modified worm-like chain model shown in equation S1.(S[2](#_ENREF_2),S[3](#_ENREF_3))

$$\frac{\Delta x}{\Delta L}=1-\frac{1}{2}\left( \frac{k_{b}T}{FP} \right)^{\frac{1}{2}}+ \frac{F}{S}\ldots\ldots\ldots Equation S1$$

Where *k*_b_ is the Boltzmann constant, *T* is absolute temperature, *P* is the persistent length (50.8 nm)(S[4](#_ENREF_4)) and *S* is the stretching modulus (1243 pN)(S[4](#_ENREF_4)).


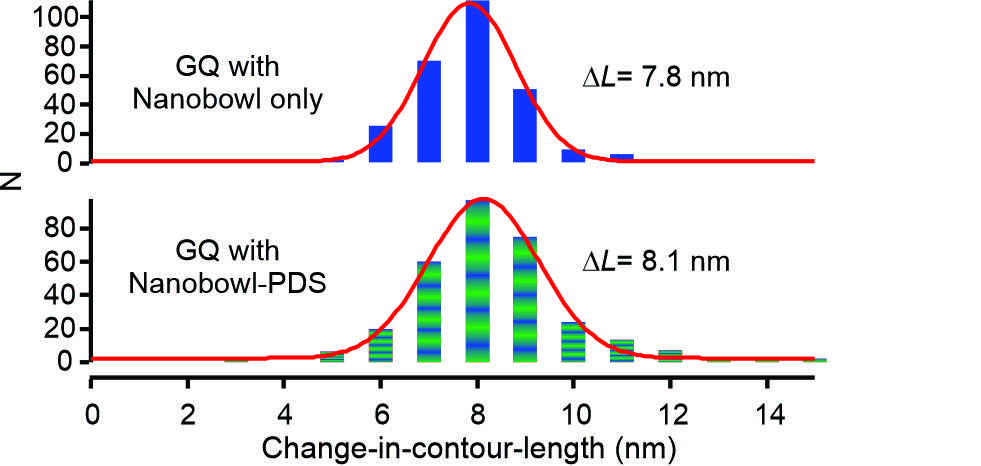


***Figure S8.*** *Change-in-contour-length (ΔL) histograms due to the unfolding of G-quadruplexes inside nanobowl (blue) and inside the nanobowl with PDS (blue and green stripes) in 20 mM Tris buffer (pH 7.8) supplemented with 10 mM MgCl_2_, 1 mM EDTA, and 100 mM KCl at 23 °C. The red curves indicate Gaussian fitting.*

# **Percentage feature formation**


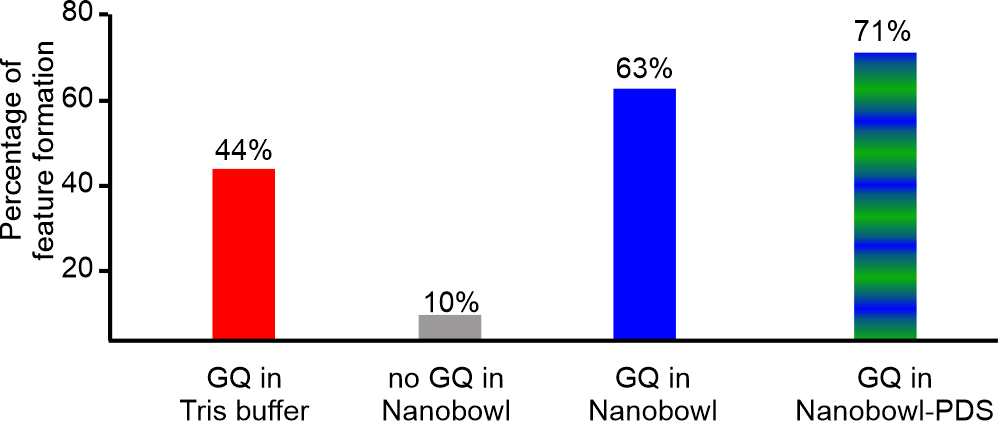


***Figure S9.*** *Bar diagrams of the percentage unfolding features for the G-quadruplex (GQ) in the Tris buffer (red, “GQ in Tris buffer”), for the scrambled sequence (Figure S7) inside nanobowl (grey, “no GQ in Nanobowl”), for the GQ inside nanobowl (blue, “GQ in Nanobowl-PDS”), and for the GQ inside nanobowl labelled with PDS (blue and green stripes) in 20 mM Tris buffer (pH 7.8) supplemented with 10 mM MgCl_2_, 1 mM EDTA, and 100 mM KCl at 23 °C.*

# **Expected change-in-contour-length (Δ*L*)**

The expected change-in-contour-length (Δ*L*) during the unfolding of a structure was calculated by the equation S2,

Δ*L = N* × *L*_nt_ – *x*…………………. (Equation S2)

where *N* is the number of nucleotides involved in the structure, *L*_nt_ is the contour length of each nucleotide (0.42 nm)([5](#_ENREF_5),[6](#_ENREF_6)), and *x* is the end-to-end distance of the hairpin structure (2 nm). 21 nucleotides (nts) (5′-GGGTTAGGGTTAGGGTTAGGG) are involved for the formation of G-quadruplex. Hence, the expected Δ*L* for the unfolding of G-quadruplex is Δ*L* = 21 × 0.42 nm – 1 nm = 7.8 nm.

# **Calculation of the change in free energy of unfolding (Δ*G*_unfold_)**

Jarzynski’s equality equation (Equation S3)(S[7](#_ENREF_7)) was used to calculate the change in free energy of unfolding (Δ*G*_unfold_) using the work associated with unfolding events.

Δ*G*_unfold_ = -*k_B_*T$\ln\sum_{i=1}^{N} \frac{1}{N} exp(-\frac{W_{i}}{k_{B}T})$………..(Equation S3)

where *k_B_* is the Boltzmann constant, *T* is the absolute temperature, *N* is the number of repetitions in the experiment, and *W* is the non-equilibrium work done to unfold the G-quadruplex. Work done was calculated using the equation S4,

W*_i_* = $\sum_{i=1}^{N} F_{i}\Delta x_{i}$ ………… (Equation S4)

where *F* and $\Delta x$ are the unfolding force and the change in end-to-end distance due to mechanical unfolding of the structure, respectively.

From the literature (S[8](#_ENREF_8)), associated bias of the Δ*G*_unfold_ was calculated. In brief, the lower tail of the work histogram was fitted by the Equation S5.

$p\left( W \right)\sim q\frac{Ω^{\alpha-1}}{|W-W_{c}|}\exp\left( -\frac{{|W-W_{c}|}^{\delta}}{Ω^{\delta}} \right)$………… (Equation S5)

where, W is the work, Wc indicates the gaussian center of the work histogram, q, Ω, α, and δ represent fitting parameters. The bias <B_N_> is calculated using these estimated parameters using Equation S6 or S7.

${<B}_{N}>= B_{REM}- \lambda^{\frac{1-\delta}{\delta}}(\gamma_{E}+\frac{1-\alpha-\delta}{\delta}\log\log N+\log\frac{q}{\delta}),$ (for $\lambda\ll1$) ………… (Equation S6)

$<B_{N}> = B_{REM}+\gamma_{E}-\lambda^{\frac{1-\delta}{\delta}}\left( \gamma_{E}+\frac{1-\alpha-\delta/2}{\delta}loglogN +\frac{1}{2}log\frac{{\pi q}^{2}}{2\delta(\delta-1)}+\theta^{2}+logerfc(\theta) \right), (for \lambda\leq1$) ………… (Equation S4)

where,

µ ≡ ($\delta-1)({\frac{\Omega}{\delta})}^{\frac{\delta}{\delta-1}}$, and

$\lambda\equiv(\delta-1)\frac{logN}{\mu}$,

*N* is the number of unfolding work measurements,

$\theta={(\lambda}^{\frac{1-\delta}{\delta}}-1)\frac{\surd\delta logN}{\surd2(\delta-1)}$,

$B_{REM}=D_{c}+\frac{{\mu(\lambda-\delta\lambda}^{\frac{1}{\delta}})}{\delta-1}$, where$D_{c}=\mu,$and $\gamma_{E}$ is the Euler-Mascheroni constant.

***
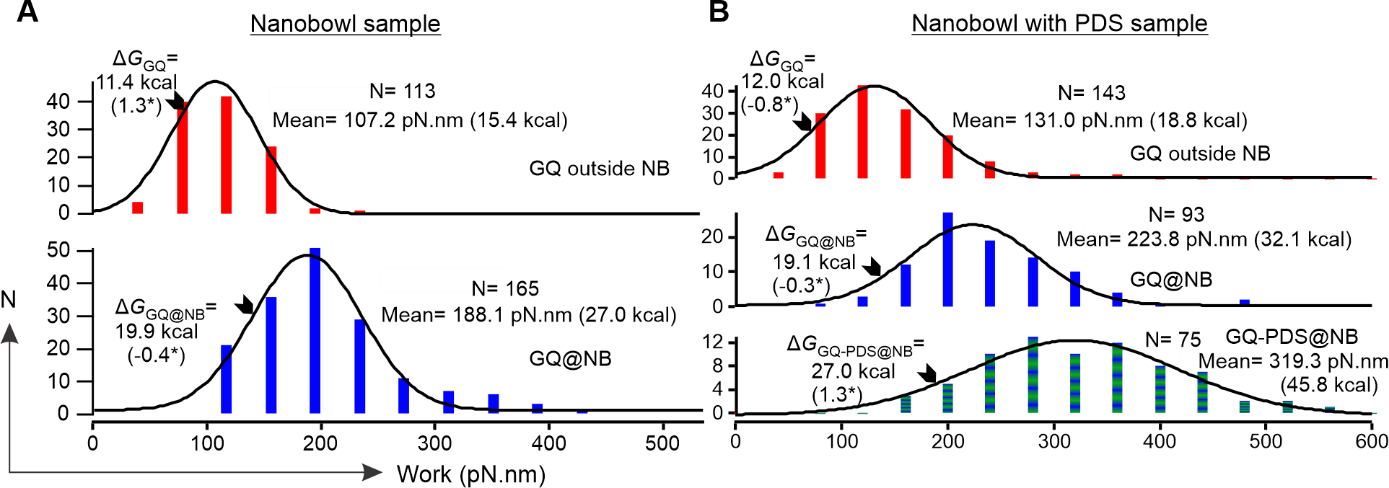
***

***Figure S10.*** *Work histograms of unfolding of G-quadruplexes (GQ) inside the nanobowl (NB) and nanobowl-PDS. Each desired unfolding force population in Figures 3D&E and 4 was first deconvoluted from the specific Gaussian peak in a multi-peak Gaussian fitting. The overlapping regions between adjacent populations were assigned to each population according to the ratio determined by the two Gaussian peaks fitted to these two populations. (S*[*9*](#_ENREF_9)*) Unfolding work was then calculated according to the Eqn S3 for these deconvoluted populations. (A) Work histograms of the telomeric G-quadruplex in the nanobowl without PDS. Work histograms outside nanobowl (red) and inside nanobowl (blue). (B) Work histograms of the telomeric G-quadruplex in the nanobowl that contains PDS. Work histograms of G-quadruplex outside the nanobowl (red), inside the nanobowl (GQ@NB, blue), and bound with the PDS inside the nanobowl (GQ-PDS@NB, blue and green stripes). The experiments were carried out in 20 mM Tris buffer (pH 7.8) supplemented with 10 mM MgCl_2_, 1 mM EDTA, and 100 mM KCl at 23 °C. The arrowheads indicate the work values equivalent of the changes in free energy of unfolding. (*) Bias for each of the change in free energy of unfolding.*

# **Hess-like cycle**


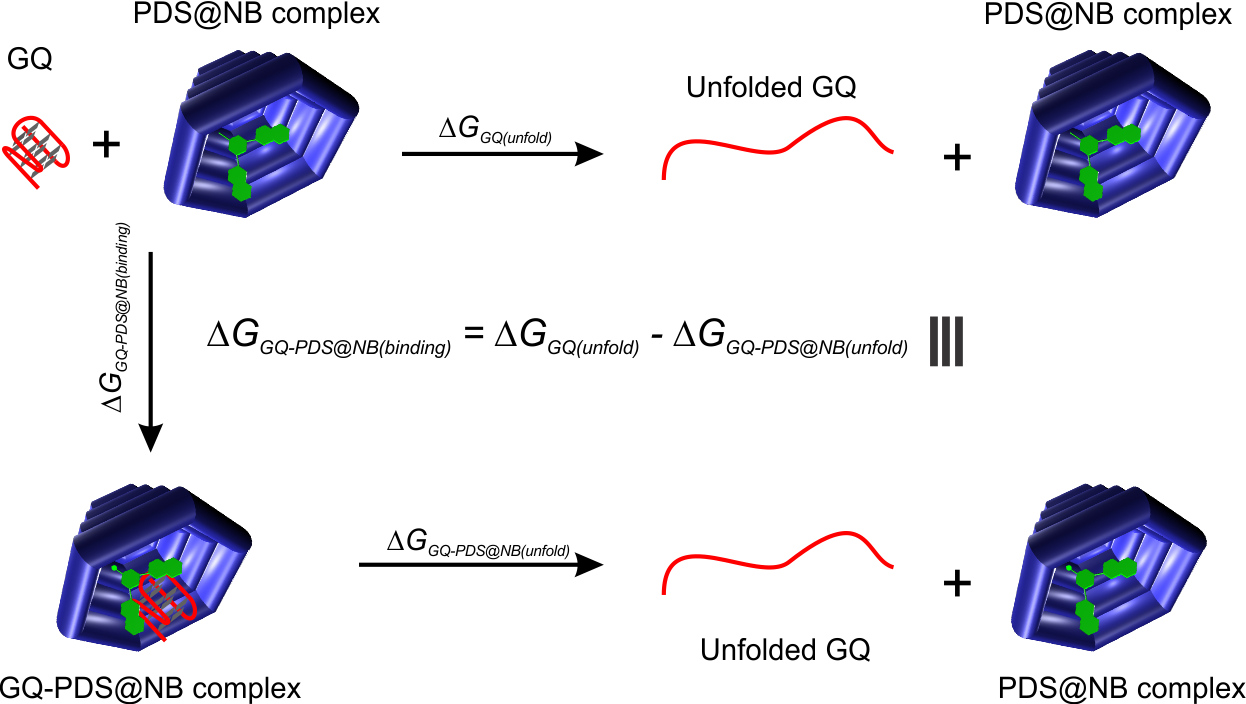


***Figure S11.*** *Hess-like cycle for the calculation of the change in the free energy of the binding between G-quadruplex (GQ) and ligand PDS inside nanobowl (NB), ΔG_GQ-PDS@NB(binding)_, inside the nanobowl. Notice ΔG_GQ-PDS@NB(binding)_ = -ΔG_GQ-PDS@NB(dissociation)_, where ΔG_GQ-PDS@NB(dissociation)_ is the change in free energy of the dissociation between the GQ and the PDS.*

# **Proximity effect versus nanoconfinement effect**


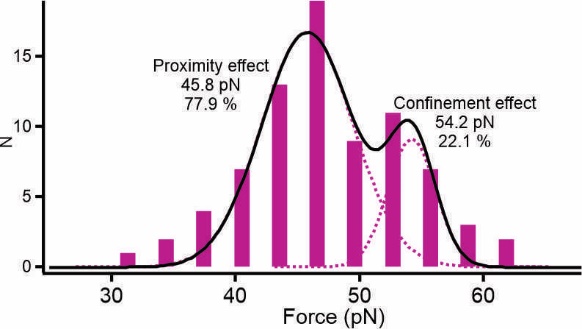


***Figure S12.*** *Deconvoluted high force populations (from Figure 3E, III) fitted with two Gaussian peaks indicate populations due to the proximity effect (~46 pN) and the nanoconfinement effect (~54 pN). Deconvolution was performed according to the literature. (S*[*9*](#_ENREF_9)*)*

**References**

S1. Rothemund, P.W.K. (2006) Folding DNA to Create Nanoscale Shapes and Patterns. *Nature*, **440**, 297-302.

S2. Baumann, C.G., Smith, S.B., Bloomfield, V.A. and Bustamante, C. (1997) Ionic effects on the elasticity of single DNA molecules. *Proc. Natl. Acad. Sci. USA.*, **94**, 6185-6190.

S3. Yu, Z. and Mao, H. (2013) Non-B DNA structures show diverse conformations and complex transition kinetics comparable to RNA or proteins ― a perspective from mechanical unfolding and refolding experiments. *Chem. Rec.*, **13**, 102-116.

S4. Dhakal, S., Cui, Y., Koirala, D., Ghimire, C., Kushwaha, S., Yu, Z., Yangyuoru, P.M. and Mao, H. (2013) Structural and mechanical properties of individual human telomeric G-quadruplexes in molecularly crowded solutions. *Nucleic Acids Res.*, **41**, 3915-3923.

S5. Mills, J.B., Vacano, E. and Hagerman, P.J. (1999) Flexibility of single-stranded DNA: use of gapped duplex helices to determine the persistence lengths of poly(dT) and poly(dA). *J. Mol. Biol.*, **285**, 245-257.

S6. Record, M.T.J., Anderson, C.F. and Lohman, T.M. (1978) Thermodynamic analysis of ion effects on the binding and conformational equilibria of proteins and nucleic acids: the roles of ion association or release, screening, and ion effects on water activity. *Quart. Rev. Biophys.*, **11**, 103-178.

S7. Jarzynski, C. (1997) Nonequilibrium Equality for Free Energy Differences. *Phys. Rev. Lett.*, **78**, 2690 - 2693.

S8. Palassini, M. and Ritort, F. (2011) Improving free-energy estimates from unidirectional work measurements: theory and experiment. *Phys Rev Lett*, **107**, 060601-060601-060605.

S9. Dhakal, S., Schonhoft, J.D., Koirala, D., Yu, Z., Basu, S. and Mao, H. (2010) Coexistence of an ILPR i-Motif and a Partially Folded Structure with Comparable Mechanical Stability Revealed at the Single-Molecule Level. *J. Am. Chem. Soc.*, **132**, 8991–8997.
